# Study of the Lipophilicity and ADMET Parameters of New Anticancer Diquinothiazines with Pharmacophore Substituents

**DOI:** 10.3390/ph17060725

**Published:** 2024-06-03

**Authors:** Daria Klimoszek, Małgorzata Jeleń, Małgorzata Dołowy, Beata Morak-Młodawska

**Affiliations:** 1 Department of Analytical Chemistry, Faculty of Pharmaceutical Sciences in Sosnowiec, Medical University of Silesia in Katowice, Jagiellońska Street 4, 41-200 Sosnowiec, Poland; d201204@365.sum.edu.pl (D.K.); mdolowy@sum.edu.pl (M.D.); 2 Department of Organic Chemistry, Faculty of Pharmaceutical Sciences in Sosnowiec, Medical University of Silesia in Katowice, Jagiellońska Street 4, 41-200 Sosnowiec, Poland; bmlodawska@sum.edu.pl

**Keywords:** lipophilicity, diquinothiazines, ADME, chromatography, phenothiazines, anticancer agents

## Abstract

Lipophilicity is one of the principal parameters that describe the pharmacokinetic behavior of a drug, including its absorption, distribution, metabolism, elimination, and toxicity. In this study, the lipophilicity and other physicochemical, pharmacokinetic, and toxicity properties that affect the bioavailability of newly synthesized dialkylaminoalkyldiquinothiazine hybrids as potential drug candidates are presented. The lipophilicity, as R_M0_, was determined experimentally by the RP-TLC method using RP18 plates and acetone–TRIS buffer (pH 7.4) as the mobile phase. The chromatographic parameters of lipophilicity were compared to computationally calculated partition coefficients obtained by various types of programs such as iLOGP, XLOGP3, WLOGP, MLOGP, SILCOS-IT, LogP, logP, and milogP. In addition, the selected ADMET parameters were determined in silico using the SwissADME and pkCSM platforms and correlated with the experimental lipophilicity descriptors. The results of the lipophilicity study confirm that the applied algorithms can be useful for the rapid prediction of logP values during the first stage of study of the examined drug candidates. Of all the algorithms used, the biggest similarity to the chromatographic value (R_M0_) for certain compounds was seen with iLogP. It was found that both the SwissADME and pkCSM web tools are good sources of a wide range of ADMET parameters that describe the pharmacokinetic profiles of the studied compounds and can be fast and low-cost tools in the evaluation of examined drug candidates during the early stages of the development process.

## 1. Introduction

Heterocyclic compounds are some of the best-known and most important structural components of drugs. Of these, nitrogen-containing heterocycles are particularly important. As the FDA (Food and Drug Administration) data show, 59% of all unique small-molecule drugs contain a nitrogen atom, and it should also be noted that 4 of the 10 most commonly used nitrogen heterocycles also contain a sulfur atom [1]. Phenothiazine is considered to be the third most commonly used six-membered nonaromatic nitrogen heterocycle and is present in 16 unique small-molecule drugs with various effects ranging from antihistaminic, sedative, and antipsychotic effects to anti-neurodegenerative (i.e., Parkinson’s and Alzheimer’s diseases) effects [1,2].

Classical phenothiazines, mainly used as neuroleptics, are substituted at position 10 with dialkylaminoalkyl groups and additionally at position 2 with small groups. These substances have significant neuroleptic, antiemetic, antihistaminic, antipruritic, analgesic, and anthelmintic effects. Continuing research in new directions on the activity of neuroleptic phenothiazines and on the modification of their structures provides information on their anticancer, antiviral (including anti-SARS-CoV-2), antibacterial, and anti-inflammatory activities, and the reversal of multidrug resistance. These substances have antioxidant and antihyperlipidemic effects [3,4,5,6,7,8,9,10].

The lipophilicity of medicinal substances has a significant impact on their ADMET parameters (absorption, distribution, metabolism, excretion, toxicity), which refer to absorption, distribution, metabolism, elimination, and toxicity. 

The assessment and consideration of the lipophilicity of medicinal substances are important during drug design, as it can have a significant impact on their pharmacokinetic properties and toxicity [11]. Medicinal substances with moderate lipophilicity tend to be better absorbed through cell membranes, which can affect their rate and absorption efficiency from the gastrointestinal tract or through the skin. Lipophilic substances can more easily penetrate cell membranes and migrate to lipid-rich tissues, which can affect their distribution in the body. Substances with increased lipophilicity may be more susceptible to metabolism in the liver through oxidation, reduction, and conjugation reactions. The impact of lipophilicity on metabolism can have consequences for pharmacological activity and toxicity. The lipophilicity of drugs can influence their excretion, as substances with increased lipophilicity can be stored in fatty tissues and exhibit a prolonged presence in the body. The lipophilicity of drugs may be related to their toxicity, as this can affect their accumulation in tissues and interactions with receptors and proteins in the body [12,13,14,15,16,17]. Therefore, to better understand the behavior of biologically active compounds, including new drug candidates, their lipophilic properties should be assessed. Theoretical and experimental methods are commonly used to describe the lipophilicity of compounds. Calculation methods are used to estimate the lipophilicity parameter quantified as P (partition coefficient) or its decimal logarithm (logP). The extensive development of chemoinformatics has an influence on the number of programs available for the online prediction (in silico) of this important parameter and other ADMET properties, and such platforms include ADMETlab, pkCSM platform, SwissADME, and MetaTox [18]. Calculation approaches are useful to rapidly predict logP values, especially during the early stages of drug development; thus, next, these values should be complemented with experimental data.

Among the experimental techniques, the classic shake-flask method and liquid chromatography play an important role in determining lipophilicity. The lipophilicity chromatographic parameters (R_M0_) that are obtained by RP-TLC (reversed-phase thin-layer chromatography) and logk_0_ and assessed by reversed-phase high-performance liquid chromatography (RP-HPLC) are commonly used to assess the lipophilic nature of compounds [19]. The standard shake-flask procedure recommended by the Organization for Economic Co-operation and Development involves the direct measurement of the partition coefficient [20]. It allows for the accurate measurement of the logP values in the range of −2 to 4 but requires relatively large amounts of pure compounds compared to other methods. The main disadvantage of this method is that it is time-consuming and requires the control of many parameters affecting the equilibrium state of the tested system, usually lasting from 1 h to 24 h [21]. Therefore, currently, most lipophilicity tests are conducted by means of chromatographic techniques. Chromatographic approaches in reversed-phase systems (RP-TLC and RP-HPLC) are the most widely used indirect methods to experimentally determine lipophilicity. Both of these chromatographic methods need a smaller amount of sample and a relatively shorter time for analysis compared to the classical shake-flask method. The obtained results are repeatable, and the accuracy of the partition coefficient values can be within ±1 unit in relation to the shake-flask value [22]. 

A comprehensive review of chromatographic procedures dedicated to the determination of the lipophilicity parameters of different drug substances as an essential tool in medicinal chemistry was performed by Soares and co-workers [23]. Taking into account the importance of lipophilicity parameters as key factors in drug chemistry, namely in the design of new drugs, the aim of this study was to assess the lipophilicity of a newly synthesized group of diquinothiazines by means of both the RP-TLC and calculation methods.

All diquinothiazines that were the subject of this study were tested early for their antiproliferative activity using cultured glioblastoma SNB-19, colorectal carcinoma Caco-2, breast cancer MDA-MB-231, and lung cancer A549 cell lines and NHDF normal fibroblasts [24]. They can therefore be considered as bioactive compounds. Most of the compounds were very active against at least one cancer cell line, with an IC_50_ value < 3 μM being more active than cisplatin. The most tested diquinothiazines showed higher activity against the A549 lung cancer cell line. Compounds **1**–**3**, **5**–**8**, and **11**–**14** were very active against all cancer cells. As was stated, the most active were the dimethylaminopropyldiquinothiazine **2** against the A549 cell line and the pyrrolidinylethyldiquinothiazine **8** against the SNB-19 cell line, with an IC_50_ value of 0.3 μM. The mechanism of the antiproliferative effect was examined using the RT-QPCR method. It caused a significant reduction in CDKN1A expression in the MDA-MB-231, A549, and SNB-19 tumor lines. Compound **8** markedly reduced the expression of *BCL-2* in A549 and SNB-19 and the expression of *BAX* in cancer cell lines [24].

In another study, the diquinothiazine **2** demonstrated significant in vitro anticancer activity against the human lung carcinoma A549 and non-small lung carcinoma H1299 lines and protective potential for the healthy cell lines BEAS-2B and NHDF. Using the 72 h MTT, strong cytotoxic activity was observed in the viability test (Promega). The weak lethal effect observed in NHDF or BEAS-2B cells at IC_50_ doses against A549 or H1299 cells confirms promising cancer selectivity. The cell cycle revealed that substance **2** activated the necrosis phase [25].

**Figure 1:**
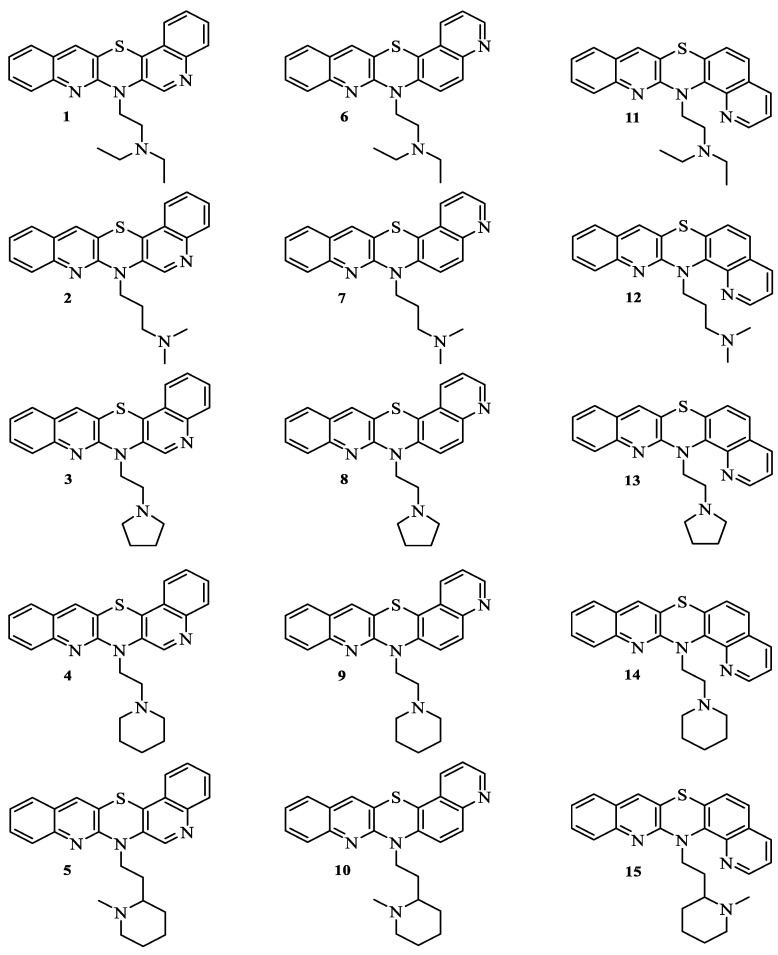
Structures of angularly condensed *N*-dialkyloaminoalkylodiquinothiazines **1**–**15**.

Continuing from our previous studies, the purpose of this work was to determine the lipophilicity parameters of fifteen newly developed anticancer, angularly condensed diquinothiazines, **1**–**15**, with pharmacophore dialkylaminoalkyl substituents using combined computational and chromatographic approaches as logP_calcd_, R_M0_, and logP_TLC_ (Figure 1). The full spectral characteristics of these compounds and their synthesis have been well described previously [24]. The current work aimed to discuss the influence of the nature of the substituents and the method of condensation of rings in a five-ring molecule system on the value of lipophilicity indices, determined by both calculation and RP-TLC methods, as well as on other drug-likeness and ADME properties predicted by in silico studies that are key for describing the pharmacokinetic behavior of these drugs. 

The usefulness of the RP-TLC technique as well as logP predictions using different computational software for the design of promising drug candidates belonging to the studied diquinothiazines with pharmacophore dialkylaminoalkyl substituents was evaluated.

## 2. Results

### 2.1. Lipophilicity Studies

Both the computational and chromatographic values of the lipophilicity parameters logP_calcd_, R_M0_, and logP_TLC_ were determined for fifteen 7- and 14-substituted angularly condensed diquinothiazines with pharmacophore dialkylaminoalkyl substituents on the thiazine nitrogen atom. The tested diquinothiazines were divided into three groups, differing in the way the quinoline rings are connected to the 1,4-thiazine ring: 7-substituted diquino[3,2-b;3′,4′-e]thiazines, **1**–**5**; 7-substituted diquino[3,2-b;6′,5′-e]thiazines, **6**–**10**; and 14-substituted diquino[3,2-b;8′,7′-e]thiazines, **11**–**15** (Figure 1).

Firstly, the computational method was chosen to determine the lipophilicity parameters of the studied compounds. For this purpose, popular computational programs were used based on various mathematical algorithms available on the following platforms: SwissADME [26], pkCSM [27], Molinspiration [28], and the ChemDraw Ultra program [29].

The calculated logP values (logP_calcd_) for the angularly fused diquinothiazines **1–15** are shown in Table 1 and differed depending on the substituents on the thiazine nitrogen atom, the shape of the five-ring diquinothiazine system, and on the calculation program.

The next step of our study focused on determining the more reliable lipophilicity parameters of the tested compounds. In order to obtain the relative lipophilicity, expressed as R_M0_, the chromatographic behavior of the fifteen tested diquinothiazines, **1**–**15**, was investigated under proper RP-TLC conditions. RP-18 plates were used as the stationary phase, while organic modifiers containing acetone were used as the mobile phase. The linear relationship between R_M0_ and acetone concentration was determined on the basis of Equation (2) (Table 2). In addition to this, thanks to the relationship observed between R_M0_ and the slope of these linear plots (b), the lipophilicity parameter C_0_ was also determined (Table 2).

Then, the relative lipophilicity parameter R_M0_ was converted to an absolute value lipophilicity parameter, logP_TLC_, using a calibration curve determined under the same measurement conditions for a set of standards, **I**–**V**, with the literature values of logP_lit_ in the range of 1.21–6.38 (Table 3). The obtained values of the R_M0_ coefficient of the tested compounds were in the range of 3.01–3.83. The correlation between the logP_lit_ values and the experimental R_M0_ values for standards **I**–**V** gave the following calibration equation:

log P_TLC_ = 1.2838 R_M0_ + 0.2138

(r = 0.9967; s = 0.1920; F = 459.32; *p* < 0.001)

The standard curve equation was used to obtain the logP_TLC_ parameter for compounds **1**–**15**, and the results are presented in Table 4.

### 2.2. Molecular Descriptors

For all tested compounds, **1**–**15**, selected molecular descriptors such as molar mass (M), molar volume (V_M_), molar refraction (Ref_M_), and surface area were calculated to check how they correlate with the experimentally determined lipophilicity parameter R_M0_ (Table 5).

### 2.3. In Silico ADME Prediction

Physicochemical parameters used to predict drug-like properties, i.e., Lipinski’s rule of five and the Ghose, Veber, Egan, and Muegge rules, were also calculated (Table 6).

ADME parameters were obtained using the pkCSM and PreADMET servers (Table 7, Table 8 and Table 9). As shown in Table 7, Table 8 and Table 9, the compounds studied showed significant differences in the molecular descriptors as well as in their ADME parameters.

#### 2.3.1. Absorption

The potential absorption of 15 tested angularly condensed diquinothiazines, **1–15**, was evaluated using the parameters of water solubility, Caco-2 cell permeability (human colon adenoma cells), absorption in the human intestine, and skin permeability, obtained using the pkCSM platform. The obtained results are summarized in Table 7. Water solubility was measured using the logS parameter (S—solubility, expressed in mol/L). When it comes to the absorption of administered drugs, the most frequently used method to determine this parameter is to test the permeability of potential drugs through a monolayer of Caco-2 cells. This is due to the similarity in structure and function of Caco-2 cells to the human intestinal epithelium. Since the main site of absorption of an orally administered drug is usually the intestine, it is important to determine the amount of the compound that is absorbed here. This method predicts the percentage of the compound that was absorbed. The skin permeability parameter is also significant for the effectiveness of some products. This parameter is expressed as logKp, and a compound is considered to have relatively low skin permeability if logKp > −2.5 [33,34].

#### 2.3.2. Distribution

The potential distribution of the tested diquinothiazines **1**–**15** was assessed using the parameters of VDss, fraction unbound, BBB permeability, and CNS permeability, obtained using the pkCSM platform. The results obtained are summarized in Table 8. The volume of distribution (VDss) provides an indication of the distribution of the drug in the body and is a pharmacokinetic parameter representing the volume into which the dose of drug would have to be distributed to give rise to the same concentration observed in the blood plasma. A low VDss indicates high water solubility or high plasma protein binding because more of the drug remains in the plasma; a high VDss suggests significant concentration in tissues, for example, due to tissue binding or high lipid solubility [35,36]. According to the model used in the pkCMS software, VDss is considered low if log VDss < −0.15 and high if log VDss > 0.45.

Fu (unbound fraction) is also an important pharmacokinetic parameter because it affects various factors of drug effectiveness and side effects (including glomerular filtration in the kidneys, total clearance, and hepatic metabolism). Therefore, it is important to accurately predict Fu during drug development. Unbound drugs in plasma may exhibit pharmacological activity by interacting with targets such as proteins, enzymes, receptors, and channels; hence, the plasma unbound fraction (Fu) of a drug is an important factor in determining drug efficacy [37].

When considering a substance as a drug candidate, it is also important to determine the extent to which it will cross the blood–brain barrier. This parameter is measured in vivo as logBB, the logarithmic ratio of brain to plasma drug concentrations. According to the computational model used in pkCSM, if the value of the logBB parameter is greater than 0.3, the substance crosses the blood–brain barrier, while if logBB is lower than −1, the substance is distributed to the brain to a small extent [38].

#### 2.3.3. Excretion and Toxicity

The potential excretion and toxicity of the tested diquinothiazines **1**–**15** were evaluated using the parameters of total clearance, maximum tolerated dose, acute oral toxicity of rats, *Tetrahymena pyriformis* toxicity, and minnow toxicity, obtained using the pkCSM platform. The results obtained are summarized in Table 9. The expected total clearance, expressed as log (ml/min/kg), is the volume of plasma completely cleared of the drug per unit of time by the organ eliminating the drug from the body. Knowledge of this parameter is necessary to determine the maintenance dose of the drug [39]. The maximum recommended tolerated dose (MRTD) allows us to initially determine the toxic dose of a chemical substance to humans. The model used in pkCSM was developed using 1222 experimental data points from human clinical trials. The obtained results of the MRTD parameter values are given as log (mg/kg/day). Calculating this parameter is helpful in determining the maximum recommended starting dose for drugs under investigation. If the calculated value is less than or equal to 0.477 log (mg/kg/day), it is considered low, and if it is higher, it is considered high. A model based on *Tetrahymena pyriformis*, a protozoal bacterium whose toxicity is often considered a toxic endpoint, was used to determine in silico toxicity in the pkCSM program. This method was built using the concentrations of 1571 compounds required to inhibit 50% of growth. This parameter is designated as plGC_50_ (negative logarithm of the concentration required to inhibit 50% growth in log µg/L), and a compound can be assessed as toxic if the value of this parameter is calculated to be greater than −0.5 log µg/L. The minnow toxicity parameter is based on measurements of the LC_50_ parameter, i.e., the concentration of the substance necessary to cause the death of 50% of flathead minnows, which were used as an animal model in this study. The model was based on tests of the LC_50_ parameter for over 550 substances. According to this model, an LC_50_ value below 0.5 mM (logLC_50_ < −0.3) is considered to indicate high acute toxicity.

## 3. Discussion

It is known that lipophilicity is one of the most frequently studied physicochemical parameters of new substances that are drug candidates [19,40]. It is most often defined to support quantitative structure–activity relationships (QSARs), including absorption, distribution in tissues, and drug transport across the biological barrier [41,42,43]. This parameter is characterized by the distribution of a dissolved substance in two-phase liquid–liquid or solid–liquid systems. In silico methods have also been proposed to assess lipophilicity. Several programs have been developed to calculate the value of the logP parameter [26,27,28,29]. It is still important to use experimental methods because most programs determining the logP parameter calculate lipophilicity using atomic methods that do not take into account some structural parameters. There may be discrepancies between the calculated data and those determined experimentally, which can be explained by the fact that newly synthesized drug candidates may contain substructures or heterocycle systems that are not covered in the software development training set [44,45].

**Figure 2:**
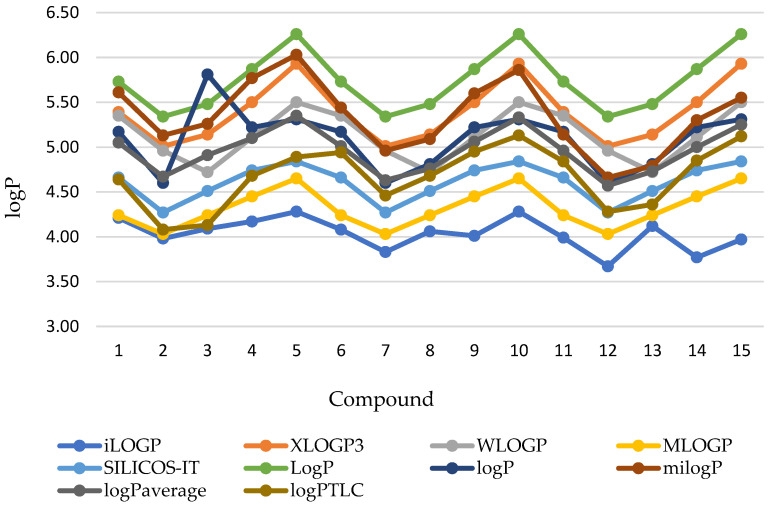
Comparison of the theoretical lipophilicity parameters (the logP values) of the examined angularly condensed *N*-dialkyloaminoalkyldiquinothiazines **1**–**15**.

The present research on lipophilicity began with in silico analyses using various computer programs available on the SwissADME [26], pkCSM [27], and Molinspiration [28] platforms and the ChemDraw program [29]. These platforms use various mathematical modules described on the website of the above-mentioned servers. The obtained results of the calculated lipophilicity are within a wide range of values. This is most likely due to differences in the calculation models. The comparison of the chromatographic parameters (logP) of the tested angularly condensed *N*-dialkylaminoalkyldiquinothiazines **1**–**15** is shown in Figure 2. As observed (Table 1), the lowest value of the logP_calc_ parameter was obtained for the 14-(3′-dimethylaminopropyl)diquino[3,2-b;8′,7′-e]thiazine **12** according to calculations with the iLOGP program (SwissADME) and the highest one was obtained for all three isomeric diquinothiazines **5**, **10**, and **15** with a piperidinylethyl substituent based on calculations with the logP program (pkCSM). This program also calculated the same values of the logP parameter for the remaining isomers (i.e., for diquinothiazines **1**, **6**, and **11**, logP = 5.17; for **2**, **7**, and **12**, logP = 4.60; for **3**, **8**, and **13**, logP = 4.81; and for **4**, **9**, and **14**, logP = 5.22).

**Figure 3:**
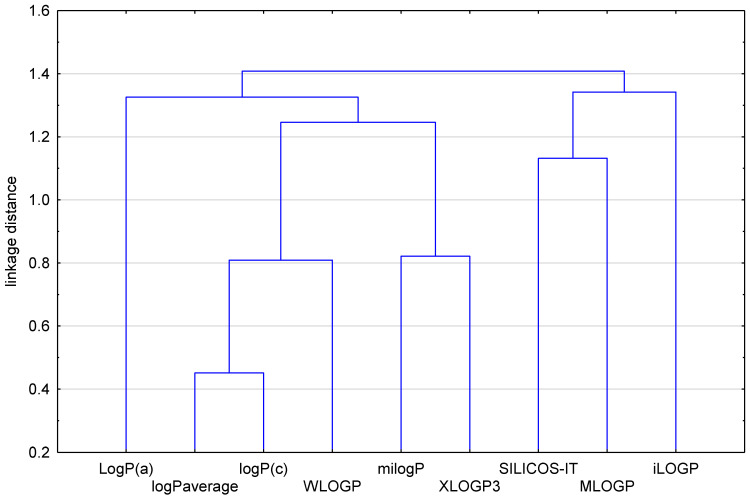
Dendrogram of partition coefficients of the examined angularly condensed *N*-dialkyloaminoalkyldiquinothiazines **1**–**15**.

**Figure 4:**
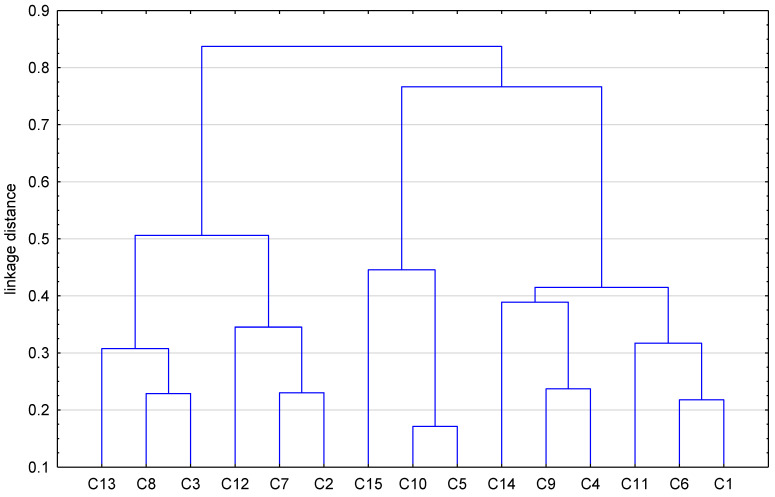
Dendrogram of the examined angularly condensed *N*-dialkyloaminoalkyldiquinothiazines **1**–**15** based on their partition coefficients.

In order to compare all obtained theoretical values of the partition coefficient of the examined drugs expressed in the form of logP, and to then estimate the lipophilic character of the studied *N*-dialkyloaminoalkyldiquinothiazines, a chemometric approach, i.e., cluster analysis (CA) with Euclidean distance, was conducted. The results are presented in Figure 3 and Figure 4.

Figure 3 shows the dendrogram of all the theoretical lipophilicity parameters of the examined angularly condensed *N*-dialkyloaminoalkyldiquinothiazines **1**–**15**. The first cluster contains the partition coefficients LogP^a^, logP_average_, logP^c^, WLOGP, milogP, and XLOGP3. In the second cluster, the rest of the computed partition coefficients are placed: SILICOS-IT, MLOGP, and iLOGP. The reason for these differences is the prediction power of each software. This grouping of partition coefficient values shows the biggest similarity (the smallest distance on the dendrogram) of logP^c^ with the logP_average_ value calculated on the basis of all theoretical logP values. Therefore, the parameter logP^c^, may be a good alternative for estimation of the lipophilicity of the examined group of N–dialkyloaminoalkyldiquinothiazines.

Figure 4 shows the similarity analysis of the examined compounds based on their partition coefficients. According to the computed lipophilicity parameters (the logP values), the studied compounds can be divided into two groups based on variation in their structural particularities, i.e., depending on the kind of substituent that may influence the lipophilic character of these compounds. The first group consists of the compounds C13, C8, and C3 in the first subgroup, with C12, C7, and C2 in the second subgroup. Similarly, the second group is divided into two smaller visible subgroups, with C15, C10, and C5 in the first subgroup and C14, C9, C4, C11, C6, and C1 in the second subgroup.

The cluster analysis shown in Figure 4 confirms the greatest similarity in lipophilic character of derivatives C10 and C5.

In the next steps of this study, the calculated logP values using different computational methods, summarized in Table 1, were correlated with the experimental lipophilicity parameter R_M0,_ presented in Table 2. The correlation coefficients and the equations obtained for the correlation of the R_M0_ parameter with the logP values obtained by means of an appropriate program are summarized in Table 10, which shows the strongest relationships between logP and R_M0_.

In order to obtain the linear equations listed in Table 10, RP-TLC was chosen as the method for experimental determination of the lipophilicity parameter of the tested diquinothiazines **1**–**15**. Using this technique, the relative lipophilicity parameter R_M0_ was obtained. It was then converted to the relative parameter logP_TLC_ (as described in Section 2 and Section 4). For all investigated derivatives, in a wide range of organic modifier concentrations in the mobile phase, high values of correlation coefficients (r = 0.99) made it possible to determine the lipophilicity parameter R_M0_ by extrapolation (Table 2). The analysis of the chromatographic parameter R_M0_ of the examined angularly condensed *N*-dialkyloaminoalkyldiquinothiazines **1**–**15** presented in Table 2 shows that they are relatively smaller compared to the theoretical logP values obtained by means of different computer software and their mean value (logP_average_). Generally, a difference in the obtained R_M0_ within one unit to logP was noted in the case of all chromatographic descriptors. This fact confirms that calculation approaches are useful only for the rapid prediction of logP values during the first stage of study of new drug candidates, after which they should be complemented with experimental data such as chromatographic descriptors. Of all the algorithms, iLogP showed the biggest similarity to the chromatographic value, especially for compounds **2**, **7**, **11**, **12**, **14**, and **15**. This shows the potential utility of iLogP for the rapid estimation of the lipophilic character of these compounds.

**Figure 5:**
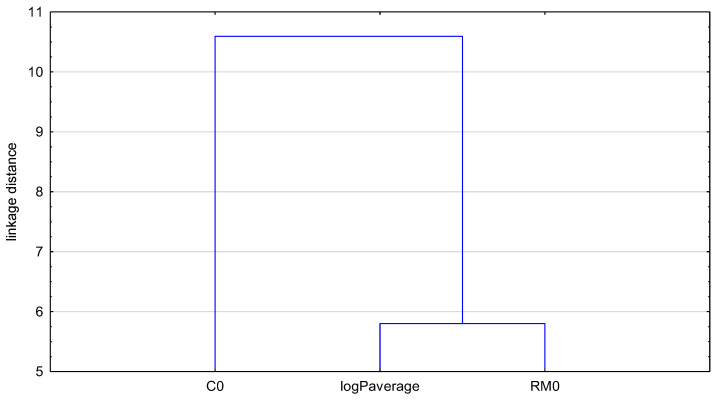
Dendrogram of theoretical (logP_averge_) and chromatographic parameters (R_M0_, C_0_) of examined angularly condensed *N*-dialkyloaminoalkyldiquinothiazines **1**–**15**.

In the next stage, satisfactory relationships between the R_M0_ values and b (slope) values of the linear equations allowed the calculation of the next lipophilicity parameter, namely C_0_. The results of these data confirm that all compounds studied belong to a congeneric group. However, as shown in Figure 5, the dissimilarity of the C_0_ values with the partition coefficients as well as the chromatographic parameter (R_M0_) indicates that both parameters cannot be fully replaced by C_0_.

The linear equations obtained between the theoretical partition coefficients and chromatographic parameters of lipophilicity of the tested compounds **1**–**15** (Table 10) confirmed the usefulness of iLOGP, XLOGP3, WLOGP, MLOGP, LOGP, miLOGP, logP_average_, and SILICOS-IT for predicting the experimental value of this parameter in the form of R_M0_.

Analyzing the experimentally obtained values of the logP_TLC_ parameter, it can be observed that in each of the three groups of isomeric angularly condensed diquinothiazines, the lowest lipophilicity was found in derivatives with a dimethylaminopropyl substituent (**2**, **7**, and **12**), while the highest was found in derivatives with an *N*-methylpiperidine substituent (**5**, **10**, and **15**). Comparing the three tested groups of isomeric angularly condensed diquinothiazines, it can be concluded that the compounds with the 7-substituted diquino[3,2-b;3′,4′-e]thiazine structure, **1–5**, are characterized by the lowest lipophilicity, while those with the 7-substituted diquino[3,2-b;6′,5′-e]thiazine structure, **6–10**, show the highest values of the logP_TLC_ parameter.

Selected molecular descriptors were also calculated for all tested diquinothiazines. The values are presented in Table 5. As shown by the results in Table 5, the same values of these parameters were obtained for isomeric angularly condensed diquinothiazines with the same substituents. The results obtained correlated with the experimentally obtained R_M0_ parameter (Table 11). Good correlations were obtained for all descriptors (r ≥ 0.8). Correlations in subgroups of individual isomers resulted in improved correlations.

### In Silico ADME Prediction

Potential drug candidates usually have similar physicochemical properties. Therefore, based on analysis of the simple molecular properties of existing drugs and/or drug candidates, simple filters defining acceptable limits for these properties are being developed. In 2007, Lipinski proposed the ‘Rule of Five’ [46], the most famous drug similarity filter which defines four rules that determine whether a molecule is well absorbed or not after oral administration. These parameters include molecular weight (MW ≤ 500), octanol/water partition coefficient (ClogP ≤ 5), number of hydrogen bond donors (HBD ≤ 5), and number of hydrogen bond acceptors (HBA ≤ 10). If the relationship violates two or more bases, it may not be active when administered orally. Data in the literature indicate that 85.4% of FDA-approved drugs meet the rule of five [47]. The results obtained on the SwissADME platform show that all tested diquinothiazines meet Lipiński’s rule.

Since then, various principles have been developed similar to the Rule of Five. For example, after parsing a file of 6304 molecules in a database, Ghose et al. found that over 80% of compounds from the Comprehensive Medicinal Chemistry (CMC) database meet the following qualification ranges: AlogP between −0.4 and 5.6, MW between 160 and 480, molar refractive index from 40 to 130, and integer atoms from 20 to 70 [48]. From the current group of 15 tested diquinothiazines, according to the SwissADME platform, derivatives with piperidine (**4**, **9,** and **14**) and *N*-methylpiperidine (**5**, **10**, and **15**) substituents do not meet this rule because their molar refractive index is greater than 130. However, the value of this parameter obtained with the program Chem3D is less than 130 (Table 5).

All tested substances, **1**–**15**, also meet Veber and Egan’s rules. According to Veber’s rule, compounds that satisfy only the following two criteria will most likely have good oral bioavailability in rats: have 10 or fewer rotatable bonds and a polar surface area equal to or less than 140 Å^2^ (or 12 or fewer hydrogen bond donors and acceptors). This may be because a reduced polar area correlates better with an increased permeation rate than lipophilicity, and an increased number of rotatable bonds has a negative effect on the permeation rate [49,50]. In turn, Egan’s rule is based on two descriptors: PSA and AlogP98.

It has been shown that molecular weight, although often used in passive absorption models, is unnecessary because it is already a component of both PSA and AlogP98. Following extensive model validation on hundreds of known orally administered drugs, “drug-like” molecules that have been tested for Caco-2 cell permeability have shown a good rate of successful predictions (74–92%) [51]. However, none of the tested substances meet Muegge’s rule due to the calculated XLOGP value being greater than 5.

The bioavailability score calculated using the SwissADME platform for all tested substances was 0.55. Substances with a bioavailability score ≥ 0.55 are considered very well absorbed by the body. A bioavailability score of 0.55 and a TPSA score between 60 and 140 Å^2^ indicate optimal absorption [52].

When developing new drugs, it is also important to determine and evaluate ADME properties (absorption, distribution, metabolism, and excretion). Poor ADME properties are a significant cause of failure and increased drug design costs [53]. It was considered acceptable to determine these parameters using in silico methods at the early stages of substance evaluation for drug suitability. This allows, without incurring high research costs, to eliminate compounds with undesirable ADME properties at an early stage of research [54]. For the discussed substances, selected parameters responsible for absorption (water solubility, Caco-2 permeability, intestinal absorption, and skin permeability), distribution (VDss, unbound fraction, BBB permeability, and CNS permeability), and excretion and toxicity (total clearance, max. tolerated dose, oral rat acute toxicity, oral rat chronic toxicity, *Tetrahymena pyriformis* toxicity, and minnow toxicity) were also determined in silico. The pkCSM platform was used to determine these parameters. All 15 tested compounds are characterized by poor solubility in water due to their chemical structure (five six-membered fused rings and no hydrophilic groups). The obtained parameter values range from −5.871 for diquinothiazine **1** to −3.965 for diquinothiazine **13**. Caco-2 permeability obtained using the pkCSM platform is given as the logarithm of the apparent permeability coefficient (log Papp). Compounds with a predicted log Papp at 10^−6^ cm/s greater than 0.9 are considered to have high Caco-2 permeability. According to the analysis results, all the synthesized compounds show high Caco-2 cell permeability (1.003–1.226). The calculated absorption values in the human intestine show that all compounds have a similar, very high probability of intestinal absorption (92.24–96.51%). The lowest values were obtained for derivatives with the 7-substituted diquino[3,2-b;3′,4′-e]thiazine structure, i.e., **1**–**5**. For the other two groups of isomers, the values were similar, which shows the influence of the shape of the molecule on this parameter. The calculated skin permeability values are in the range of −2.744 to −2.694 and indicate poor permeability (Table 7). Almost all tested angularly condensed diquinothiazines can be substrates for P-glycoprotein (except for substance **1**), and all of them are predicted to inhibit P-glycoproteins I and II (Table S1).

VDss is a pharmacokinetic parameter that determines half-life and represents the degree of drug distribution in tissues. The predicted log VDss values ranged from 0.86 to 1.37, which means that the compounds have a high constant distribution in plasma and tissues. The unbound fraction in plasma (Fu) is also an important pharmacokinetic parameter which determines the amount of drug that is “free” in the plasma, and therefore the fraction capable of diffusing from the plasma into tissues. From the results obtained for the tested diquinothiazines **1**–**15**, the unbound fraction had low values ranging from 0.189 to 0.268. The BBB permeability parameter was also determined using the pkCSM platform. The blood–brain barrier controls the transfer of essential substances needed for a proper functioning brain and participates in the removal of cellular metabolites and toxins. The expected permeability through the blood–brain barrier for the tested substances ranged from 0.357 to 0.572, which means that they will pass through the BBB barrier. All tested diquinothiazines may also be able to penetrate the central nervous system because the logPS parameter values obtained for them were in the range of −1.304 to −1.503, and substances with logPS > −2 are considered to penetrate the CNS, while those with logPS < −3 do not penetrate the CNS (Table 8).

The possible interaction of the tested diquinothiazines with cytochrome P450 was also calculated. The obtained results show that all tested substances may be CYP1A2 inhibitors, and almost all of them may be CYP2D6 (except substances **3** and **4**) and CYP3A4 (except substance **2**) inhibitors; however, none of the tested compounds may be CYP2C9 inhibitors (Table S1). Although the abundance of CYP3A4 is poor, it contributes to nearly 50% of drug metabolism, so drug–drug interactions need to be considered in the subsequent development of compounds.

The excretion and toxicity parameters determined in silico are presented in Table 9. Total clearance of the tested diquinothiazines ranged from 0.526 to 0.832. Total clearance measures the efficiency of drug elimination from the entire body and is useful in determining the rate of drug dosing. The maximum tolerated dose value for the tested diquinothiazines was calculated in the range from 0.188 to 0.672. A dose equal to or less than 0.477 is considered low, and such values were obtained for diquinothiazines **6**–**10**, while dose values higher than 0.477, which are considered high, were obtained for the remaining compounds tested. The values of the parameters of oral rat acute toxicity and chronic toxicity were calculated in the range from 2.272 to 3.184 and from 0.449 to 1.096, respectively. The determined values of the *Tetrahymena pyriformis* toxicity parameter were above −0.5 (0.291–0.331), which means that the compounds may be toxic, while the minnow toxicity parameter values ranged from −0.989 to 1.944. Values of this parameter of less than −0.3 may indicate the toxicity of the substance and were calculated for diquinothiazines **4**, **8**, **9**, **13**, and **14**. It was also checked whether the tested substances could be substrates of Organic Cation Transporter 2 (Renal OCT2). Renal OCT2 is a renal uptake transporter that plays an important role in renal clearance and the clearance of drugs and endogenous compounds. Diquinothiazines **8** and **9** may have this effect (Table S2). However, none of the substances discussed should show AMES toxicity or irritate the skin, but they may be hepatotoxic. Calculations using the pkCSM program also showed that the tested substances may be hERG II inhibitors but should not show any inhibition against hERG I (Table S1).

**Figure 6:**
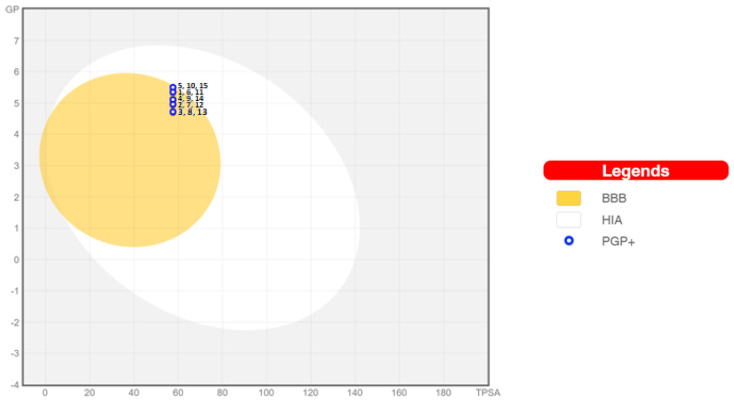
The boiled egg representation of the intestinal absorption and permeation through the blood–brain barrier for diquinothiazines **1**–**15**.

Absorption from the gastrointestinal tract and access to the brain are two pharmacokinetic parameters important to estimate at different stages of the drug discovery process. For this purpose, an estimated method of penetration into the brain or intestines has been developed—boiled egg [55]. It is an accurate predictive model that works by calculating the lipophilicity and polarity of small molecules. At the same time, penetration into both the brain and intestines is predicted based on the same two physicochemical descriptors. This computational method was also used to analyze the tested diquinothiazines **1**–**15** (Figure 6). Molecules located in the white region are expected to be passively absorbed in the gastrointestinal tract, while molecules in the “yolk” region are expected to passively cross the BBB. It can be observed that none of the analyzed compounds can be passively absorbed in the intestines, but all of them can pass through the BBB. This shows that the dosing compliance may be poor due to the inability of all the compounds to be absorbed through the intestines. Additionally, all tested compounds can become substrates for p-glycoprotein (blue points).

## 4. Materials and Methods

### 4.1. Solvents and Reference Standards

The studied compounds, i.e., 7-substituted diquino[3,2-b;3′,4′-e]thiazines **1**–**5**, 7-substituted diquino[3,2-b;6′,5′-e]thiazines **6**–**10**, and 14-substituted diquino[3,2-b;8′,7′-e]thiazines **11**–**15**, were previously designed and synthesized [24]. The chemical structures of the examined compounds are shown in Figure 1. The summary formulas, molecular weights, melting points, and physical appearances of the tested diquinothiazines **1–15** are listed in Table S3.

Analytical grade acetone (POCh, Gliwice, Poland) and TRIS (tris(hydroxymethyl)aminomethane, Fluka, Buchs, Switzerland) were used in RP-TLC studies as the mobile phase components. To prepare the calibration curve, the reference standards of five chemical compounds with the described lipophilicity parameter (logP_lit_) were used: acetanilide (**I**, 1.21 [30], POCh, Gliwice, Poland), benzoic acid (**II**, 1.87 [31], POCh, Gliwice, Poland), benzophenone (**III**, 3.18 [31], Fluka, Buchs, Switzerland), anthracene (**IV**, 4.45 [31], POCh, Gliwice, Poland), and p,p′-DDT (1,1,1-trichloro-2,2-bis(4-chlorophenyl, Merck, Darmstadt, Germany), **V**, 6.38 [32]).

### 4.2. RP-TLC Analysis

All chromatographic experiments were performed on a silica gel 60 RP-18 F_254S_ (10 cm × 10 cm) RP-TLC plate (Merck, Darmstadt, Germany). The mobile phase was prepared by mixing the respective amount of aqueous TRIS (tris(hydroxymethyl)aminomethane) buffer, pH = 7.4 (ionic strength 0.2 M), to meet physiological conditions and acetone in a range from 50 to 80% (*v*/*v*) in increments of 5%. Ethanol (96%) was used to prepare the solutions of angularly condensed diquinothiazines **1**–**15** and the standards **I**–**V** at a concentration of 2.0 mg/mL. Next, solutions (2 mL) of the analyzed compounds were applied to the plates 5 mm apart and 10 mm from the lower edge and sides of the plates. Before plate development, the chromatographic chambers were saturated with the mobile phase for 0.5 h. After the development of the plates and drying in a stream of air, the chromatograms were observed in ultraviolet light at 254 nm. Each chromatographic experiment was run in triplicate, and then mean values of the retardation factor, i.e., R_F_ values, were used to determine the R_MW_ values of the compounds.

The R_M_ values calculated from the experimental R_F_ values by using Equation (1) were linearly dependent on the concentration of acetone:

(1) $$R_{M}=\log\left(\frac{1}{R_{F}}-1\right)$$

The R_M0_ values were obtained by extrapolating to zero acetone concentration by using Equation (2):

(2) $$R_{M}=R_{M0}+b\cdot C$$

where C is the volume fraction of the organic modifier in the mobile phase and b is the change in the R_M_ value due to the 1% increase in the organic modifier in the mobile phase (associated with the specific hydrophobic surface area) [56].

Next, the chromatographic data in the form of R_M0_ and coefficient b were used to calculate the lipophilicity parameter C_0_ [56] from the following Equation (3):

(3) $$C_{0}=-\frac{R_{M0}}{b}$$

The C_0_ parameter is interpreted as the molecular hydrophobicity per unit of the specific hydrophobic surface, i.e., corresponding to the specific hydrophobic surface area of the substance in contact with the stationary phase.

### 4.3. In Silico Calculated Descriptors

Theoretical values of the partition coefficients (logP) for each compound were determined using various Internet servers such as SwissADME [26], pkCSM [27], Molinspiration [28], and the ChemDraw program [29], including iLOGP, XLOGP3, WLOGP, MLOGP, SILCOS-IT, LogP, logP, and milogP. The logP_average_ is an arithmetic mean of the values predicted by the algorithms. The software used are based on different methods of logP calculation [52,57,58]. The following lipophilicity descriptors were obtained using the web tool SwissADME: iLOGP, a physics-based method that relies on free energies of solvation in n-octanol and water calculated by the generalized-born and solvent accessible surface area (GB/SA) model developed by Daina and coworkers; WLOGP, an atomistic method stationed on fragments and topological descriptors; XLOGP3, an atomistic method including corrective factors and a knowledge-based library; MLOGP, based on topological indices and the linear relationship of structure–logP; and SILICOS-IT, a hybrid fragment/topological approach based on 27 fragments and 7 topological descriptors [26,52,57,58]. The next method used for the calculation of miLogP is a fragment-based approach developed by Molinspiration, and it calculates log P from the sum of group or fragment contributions and correction factors [28]. To compute all other descriptors, such as LogP^a^ and logP^c^, the pkCSM and ChemDraw programs were used, respectively [27,29].

The molecular descriptors and the ADME parameters were also obtained using the SwissADME and pkCSM platform [26,27].

The cluster analysis procedure was conducted using Statistica software v. 13.3. Calculations were performed on Euclidean distances and a single linkage distance [59].

## 5. Conclusions

In the search for the most promising drug-like substances, the key parameter that describes the pharmacokinetic behavior of a drug is lipophilicity. Therefore, in the present work, fifteen newly synthesized anticancer, angularly condensed diquinothiazines with pharmacophore dialkylaminoalkyl substituents were studied with the goal of predicting selected pharmacokinetic parameters influencing their ADMET properties, including the lipophilicity descriptors logP and R_M0_. Lipophilicity indices were determined experimentally by the RP-TLC method, with RP_18_ as the stationary phase and an acetone–TRIS buffer (pH 7.4) mixture as the mobile phase, as well as theoretically using computer approaches. In addition to this, the selected ADMET parameters were determined in silico using the SwissADME and pkCSM platforms and then correlated with the experimental lipophilicity parameters of the examined compounds. The results of the lipophilicity study confirm that the applied software can be useful for the rapid prediction of logP values during the first stage of study of the examined drug candidates, and next they should be complemented with experimental data such as chromatographic descriptors. Of all the algorithms used, iLogP showed the biggest similarity to the chromatographic value (R_M0_), especially for compounds **2**, **7**, **12**, **14**, and **15**. In addition to this, it was stated that both the SwissADME and pkCSM web tools are good sources of a wide range of ADMET parameters that describe the pharmacokinetic profiles of the studied compounds and can be fast and low-cost tools in the evaluation of drug candidates during the early stages of the development process.

The satisfactory linear correlation equations of the chromatographic parameter of lipophilicity (R_M0_) with the theoretical logP values and other physicochemical properties of the tested compounds, such as the molar mass, molar volume, molar refractive index, and surface area, and with the results of other pharmacokinetics parameters that are key in the ADMET profile demonstrate the utility of the computational approaches in estimating the physicochemical properties of new drug candidates in the development process.

## Figures and Tables

**Table 1:** The computed lipophilicity parameters (logP_calcd_) for diquinothiazines **1**–**15** using the following Internet databases: SwissADME [26], pkCSM ^a^ [27], Molinspiration ^b^ [28], and ChemDraw ^c^ [29].

No. of Compound	iLOGP	XLOGP3	WLOGP	MLOGP	SILICOS-IT	LogP ^a^	milogP ^b^	logP ^c^	logP_average_
**1**	4.21	5.39	5.35	4.24	4.66	5.73	5.61	5.17	5.05 (±0.60)
**2**	3.98	5.01	4.96	4.03	4.27	5.34	5.13	4.60	4.67 (±0.52)
**3**	4.09	5.14	4.72	4.24	4.51	5.48	5.26	4.81	4.91 (±0.61)
**4**	4.17	5.50	5.11	4.45	4.74	5.87	5.77	5.22	5.10 (±0.61)
**5**	4.28	5.93	5.50	4.65	4.84	6.26	6.03	5.31	5.35 (±0.71)
**6**	4.08	5.39	5.35	4.24	4.66	5.73	5.44	5.17	5.01 (±0.61)
**7**	3.83	5.01	4.96	4.03	4.27	5.34	4.96	4.60	4.63 (±0.53)
**8**	4.06	5.14	4.72	4.24	4.51	5.48	5.09	4.81	4.76 (±0.48)
**9**	4.01	5.50	5.11	4.45	4.74	5.87	5.60	5.22	5.06 (±0.63)
**10**	4.28	5.93	5.50	4.65	4.84	6.26	5.86	5.31	5.33 (±0.69)
**11**	3.99	5.39	5.35	4.24	4.66	5.73	5.14	5.17	4.96 (±0.60)
**12**	3.67	5.01	4.96	4.03	4.27	5.34	4.66	4.60	4.57 (±0.55)
**13**	4.12	5.14	4.72	4.24	4.51	5.48	4.79	4.81	4.73 (±0.45)
**14**	3.77	5.50	5.11	4.45	4.74	5.87	5.30	5.22	5.00 (±0.66)
**15**	3.97	5.93	5.50	4.65	4.84	6.26	5.55	5.31	5.25 (±0.74)

**Table 2:** Data for linear correlation (R_M_ = R_M0_ + bC) for compounds **1**–**15**.

No. of Compound	R_M0_	b	r	C_0_
**1**	3.45	−4.77	0.9932	0.7233
**2**	3.01	−4.09	0.9961	0.7359
**3**	3.05	−4.23	0.9916	0.7210
**4**	3.48	−4.89	0.9953	0.7117
**5**	3.64	−5.14	0.9980	0.7082
**6**	3.68	−4.85	0.9951	0.7588
**7**	3.31	−4.34	0.9938	0.7627
**8**	3.48	−4.60	0.9941	0.7565
**9**	3.69	−4.88	0.9962	0.7562
**10**	3.83	−5.04	0.9962	0.7599
**11**	3.60	−4.75	0.9927	0.7579
**12**	3.17	−4.25	0.9935	0.7458
**13**	3.23	−4.21	0.9907	0.7672
**14**	3.61	−4.69	0.9952	0.7697
**15**	3.82	−5.01	0.9942	0.7625

**Table 3:** **The** R_M0_ and logP_lit_ values and b (slope) and r (correlation coefficient) values of the equation R_M_ = R_M0_+ bC for standards **I**–**V**.

Lipophilicity Parameters	Standards				
	I	II	III	IV	V
logP_lit_	1.21 [30]	1.87 [31]	3.18 [31]	4.45 [31]	6.38 [32]
R_M0_	0.78	1.16	2.51	3.33	4.69
-b	0.0162	0.0247	0.0328	0.0412	0.0564
r	0.9923	0.9937	0.9971	0.9982	0.9977
logP_TLC_	1.21	1.70	3.43	4.49	6.24

**Table 4:** The experimental lipophilicity parameters (logP_TLC_ values) for compounds **1**–**15**.

No. of Compound	logP_TLC_	No. of Compound	logP_TLC_	No. of Compound	logP_TLC_
**1**	4.64	**6**	4.94	**11**	4.84
**2**	4.08	**7**	4.46	**12**	4.28
**3**	4.13	**8**	4.68	**13**	4.36
**4**	4.68	**9**	4.95	**14**	4.85
**5**	4.89	**10**	5.13	**15**	5.12

**Table 5:** The molecular descriptors for compounds **1**–**15**.

No. of Compound	Molar Mass (M) [g/mol]	Molar Volume (V_M_) [cm^3^]	Molar Refractivity (Ref_M_) [cm^3^/mol]	Surface Area [Å]
**1**	400.55	322.1	121.077	175.12
**2**	386.52	305.6	116.446	168.75
**3**	398.54	304.6	119.121	174.11
**4**	412.56	322.4	123.722	180.48
**5**	426.59	342.3	128.258	186.84
**6**	400.55	322.1	121.077	175.12
**7**	386.52	305.6	116.446	168.75
**8**	398.54	304.6	119.121	174.11
**9**	412.56	322.4	123.722	180.48
**10**	426.59	342.3	128.258	186.84
**11**	400.55	322.1	121.077	175.12
**12**	386.52	305.6	116.446	168.75
**13**	398.54	304.6	119.121	174.11
**14**	412.56	322.4	123.722	180.48
**15**	426.59	342.3	128.258	186.84

**Table 6:** The drug-likeness and ADME properties predicted by in silico studies using SwissADME.

Predicted Parameter	Compound No.				
	1, 6, 11	2, 7, 12	3, 8, 13	4, 9, 14	5, 10, 15
Physicochemical Properties					
Num. heavy atoms	29	28	29	30	31
Num. arom. heavy atoms	20	20	20	20	20
Hydrogen bond acceptors	3	3	3	3	3
Hydrogen bond donors	0	0	0	0	0
Number of rotatable bonds	5	4	3	3	3
Topological polar surface area [Å^2^]	57.56	57.56	57.56	57.56	57.56
Drug-Likeness Prediction					
Rule of Lipinski	+	+	+	+	+
Rule of Ghose	+	+	+	−(MR>130)	−(MR>130)
Rule of Veber	+	+	+	+	+
Rule of Egan	+	+	+	+	+
Rule of Muegge	−(XLOGP3>5)	−(XLOGP3>5)	−(XLOGP3>5)	−(XLOGP3>5)	−(XLOGP3>5)
Bioavailability					
Bioactivity score	0.55	0.55	0.55	0.55	0.55

**Table 7:** The absorption descriptors for compounds **1**–**15**.

No. of Compound	Water Solubility [log mol/L]	Caco-2 Permeability [log Papp in 10^−6^ cm/s]	Intestinal Absorption [% Absorbed]	Skin Permeability [log Kp]
**1**	−5.871	1.003	92.241	−2.697
**2**	−5.783	1.028	92.931	−2.701
**3**	−4.551	1.145	92.725	−2.733
**4**	−4.660	1.143	92.337	−2.733
**5**	−5.820	1.021	92.309	−2.711
**6**	−5.793	1.032	95.220	−2.694
**7**	−5.737	1.057	96.401	−2.700
**8**	−4.517	1.226	94.349	−2.723
**9**	−4.652	1.224	93.960	−2.723
**10**	−5.829	1.049	95.779	−2.712
**11**	−5.373	1.043	95.329	−2.691
**12**	−5.263	1.068	96.510	−2.697
**13**	−3.965	1.211	93.469	−2.744
**14**	−4.061	1.209	93.080	−2.743
**15**	−5.329	1.060	95.888	−2.709

**Table 8:** The distribution descriptors for compounds **1**–**15**.

No. of Compound	VDss [log L/kg]	Unbound Fraction [Fu]	BBB Permeability [log BB]	CNS Permeability [log PS]
**1**	0.986	0.259	0.478	−1.464
**2**	0.864	0.253	0.483	−1.402
**3**	0.868	0.193	0.424	−1.394
**4**	0.923	0.189	0.437	−1.381
**5**	1.041	0.255	0.535	−1.422
**6**	1.178	0.268	0.537	−1.493
**7**	1.062	0.262	0.484	−1.478
**8**	1.269	0.206	0.559	−1.318
**9**	1.328	0.200	0.572	−1.304
**10**	1.244	0.257	0.536	−1.498
**11**	1.332	0.267	0.387	−1.475
**12**	1.200	0.261	0.357	−1.483
**13**	1.170	0.200	0.425	−1.370
**14**	1.222	0.196	0.438	−1.356
**15**	1.368	0.262	0.410	−1.503

**Table 9:** The excretion and toxicity for compounds **1**–**15**.

No. of Compound	Total Clearance [log ml/min/kg]	Max. Tolerated Dose [log mg/kg/day]	Oral Rat Acute Toxicity [mol/kg]	Oral Rat Chronic Toxicity [log mg/kg bw/day]	*Tetrahymena pyriformis* Toxicity [log μg/L]	Minnow Toxicity [log mM]
**1**	0.672	0.672	2.277	0.449	0.299	1.983
**2**	0.526	0.647	2.272	0.490	0.300	1.944
**3**	0.824	0.537	2.734	0.827	0.291	−0.279
**4**	0.779	0.550	2.756	0.848	0.291	−0.396
**5**	0.599	0.568	2.363	0.542	0.291	1.736
**6**	0.714	0.293	2.361	0.773	0.327	0.423
**7**	0.584	0.258	2.312	0.807	0.331	0.597
**8**	0.832	0.211	3.157	1.074	0.300	−0.872
**9**	0.787	0.228	3.184	1.096	0.299	−0.989
**10**	0.657	0.188	2.413	0.606	0.304	0.389
**11**	0.707	0.594	2.513	0.590	0.299	0.542
**12**	0.562	0.557	2.485	0.610	0.300	0.460
**13**	0.826	0.543	2.949	1.020	0.292	−0.848
**14**	0.781	0.557	2.972	1.041	0.291	−0.965
**15**	0.634	0.483	2.588	0.663	0.291	0.252

**Table 10:** Linear correlations between the partition coefficients and the chromatographic parameters of lipophilicity of the tested compounds **1**–**15** (*p* < 0.05).

Compounds	Lipophilicity Parameter	Equation	r
**1**–**5**	iLOGP	iLOGP = 0.3877 R_M0_ + 2.8566	0.9403
**1**–**15**	XLOGP3	XLOGP3 = 1.0578 R_M0_ + 1.7233	0.8426
**1**–**5**		XLOGP3 = 1.1972R_M0_ + 1.4122	0.9384
**6**–**10**		XLOGP3 = 1.6538 R_M0_ − 0.5565	0.9427
**11**–**15**		XLOGP3 = 1.2383 R_M0_ + 1.0773	0.9577
**1**–**15**	WLOGP	WLOGP = 0.8350 R_M0_ + 2.2304	0.7683
**1**–**5**		WLOGP = 0.9794 R_M0_ + 1.8705	0.8868
**11**–**15**		WLOGP = 0.9985 R_M0_ + 1.6473	0.8919
**1**–**15**	MLOGP	MLOGP = 0.6500 R_M0_ + 2.0664	0.7840
**6**–**10**		MLOGP = 1.0545 R_M0_ + 0.5279	0.9101
**11**–**15**		MLOGP = 0.7511 R_M0_ + 1.7035	0.8795
**1**–**15**	SILCOS-IT	SILCOS-IT = 0.6630 R_M0_ + 2.3034	0.8486
**1**–**5**		SILCOS-IT = 0.7442 R_M0_ + 2.1287	0.9374
**6**–**10**		SILCOS-IT = 1.0799 R_M0_ + 0.7184	0.9892
**11**–**15**		SILCOS-IT = 0.7588 R_M0_ + 1.9588	0.9432
**1**–**15**	LogP	LogP = 1.0697 R_M0_ + 2.0243	0.8484
**1**–**5**		LogP = 1.2120 R_M0_ + 1.7050	0.9460
**6**–**10**		LogP = 1.6739 R_M0_ − 0.2866	0.9500
**11**–**15**		LogP = 1.2499 R_M0_ + 1.3788	0.9624
**6**–**10**		logP = 1.4693 R_M0_ − 0.2644	0.9877
**11**–**15**		logP = 1.0652 R_M0_ + 1.3086	0.9714
**1**–**15**	milogP	milogP = 0.9755 R_M0_ + 1.9610	0.6499
**1**–**5**		milogP = 1.2903 R_M0_ + 1.2684	0.9813
**6**–**10**		milogP = 1.7585 R_M0_ − 0.9371	0.9725
**11**–**15**		milogP = 1.3037 R_M0_ + 0.5431	0.9867
**1**–**15**	logP_average_	logP_average_ = 0.7369 R_M0_ + 2.4017	0.7854
**1**–**5**		logP_average_ = 0.8307 R_M0_ + 2.2530	0.9292
**6**–**10**		logP_average_ = 1.3129 R_M0_ + 0.2343	0.9791
**11**–**15**		logP_average_ = 0.9300 R_M0_ + 1.6601	0.9819

**Table 11:** Linear correlations between experimentally determined R_M0_ and the molecular descriptors’ values (*p* < 0.05).

Compounds	Molecular Descriptor	Equation	r
**1**–**15**	Molar mass	M = 42.779 R_M0_ + 256.508	0.8000
**1**–**5**		M = 47.859 R_M0_ + 245.773	0.8807
**6**–**10**		M = 68.869 R_M0_ + 157.161	0.9216
**11**–**15**		M = 49.570 R_M0_ + 232.152	0.8999
**1**–**15**	Molar volume	V_M_ = 45.782 R_M0_ + 160.711	0.8444
**1**–**5**		V_M_ = 52.381 R_M0_ + 145.180	0.9518
**6**–**10**		V_M_ = 69.514 R_M0_ + 69.288	0.9182
**11**–**15**		V_M_ = 53.970 R_M0_ + 131.262	0.9674
**1**–**15**	Molar refractivity	Ref_M_ = 13.202 R_M0_ + 75.913	0.8311
**1**–**5**		Ref_M_ = 14.871 R_M0_ + 72.263	0.9213
**6**–**10**		Ref_M_ = 20.942 R_M0_ + 46.375	0.9434
**11**–**15**		Ref_M_ = 15.363 R_M0_ + 68.170	0.9389
**1**–**15**	Surface area	Surface area = 19.960 R_M0_ + 109.882	0.8017
**1**–**5**		Surface area = 21.667 R_M0_ + 105.000	0.8831
**6**–**10**		Surface area = 31.142 R_M0_ + 65.010	0.9229
**11**–**15**		Surface area = 22.437 R_M0_ + 98.843	0.9021

## Data Availability

Data from the research described in the manuscript are available from the authors.

## Supplementary Materials

The following supporting information can be downloaded at: https://www.mdpi.com/article/10.3390/ph17060725/s1, Table S1: The absorption and metabolism descriptors for compounds **1**–**15**; Table S2: The excretion and toxicity descriptors for compounds **1**–**15**; Figure S1: The bioavailability radars for compounds **1**–**15**; Table S3: The summary formulas, molecular weights, melting points, and appearance of the tested diquinothiazines **1**–**15**.

## References

[1] Vitaku E., Smith D.T., Njardarson J.T. (2014). Analysis of the structural diversity, substitution patterns, and frequency of nitrogen heterocycles among US FDA approved pharmaceuticals. J. Med. Chem..

[2] Ohlow M.J., Moosmann B. (2011). Foundation Review: Phenothiazine: The seven lives of pharmacology’s first lead structure. Drug Discov. Today.

[3] Edinoff A.N., Armistead G., Rosa C.A., Anderson A., Patil R., Cornett E.M., Murnane K.S., Kaye A.M., Kaye A.D. (2022). Phenothiazines and their evolving roles in clinical practice: A narrative review. Health Psychol. Res..

[4] Posso M.C., Domingues F.C., Ferreira S., Silvestre S. (2022). Development of phenothiazine hybrids with potential medicinal interest: A Review. Molecules.

[5] Pluta K., Morak-Młodawska B., Jeleń M. (2011). Recent progress in biological activities of synthesized phenothiazines. Eur. J. Med. Chem..

[6] Spengler G., Csonka Á., Molnár J., Amaral L. (2016). The anticancer activity of the old neuroleptic phenothiazine-type drug thioridazine. Anticancer Res..

[7] Zubas A., Ghinet A., Farce A., Dubois J., Bîcu E. (2023). Phenothiazineand carbazole-cyanochalcones as dual inhibitors of tubulin polymerization and human farnesyltransferase. Pharmaceuticals.

[8] Mosnaim A.D., Ranade V.V., Wolf M.E., Puente J., Valenzuela A.M. (2006). Phenothiazine molecule provides the basic chemical structure for various classes of pharmacotherapeutic agents. Am. J. Ther..

[9] Chuang S.T., Papp H., Kuczmog A., Eells R., Condor Capcha J.M., Shehadeh L.A., Jakab F., Buchwald P. (2022). Methylene blue is a nonspecific protein–protein interaction inhibitor with potential for repurposing as an antiviral for COVID-19. Pharmaceuticals.

[10] Boyd-Kimball D., Gonczy K., Lewis B., Mason T., Siliko N., Wolfe J. (2019). Classics in chemical neuroscience: Chlorpromazine. ACS Chem. Neurosci..

[11] Ginex T., Vazquez J., Gilbert E., Herrero E., Luque F.J. (2019). Lipophilicity in drug design: An overview of lipophilicity descriptors in 3D-QSAR studies. Future Med. Chem..

[12] Johnson T.W., Dress K.R., Edwards M. (2009). Using the Golden Triangle to optimize clearance and oral absorption. Bioorg. Med. Chem. Lett..

[13] Johnson T.W., Gallego R.A., Edwards M.P. (2018). Lipophilic efficiency as an important metric in drug design. J. Med. Chem..

[14] Ditzinger F., Price D.J., Ilie A.R., Köhl N.J., Jankovic S., Tsakiridou G., Aleandri S., Kalantzi L., Holm R., Nair A. (2019). Lipophilicity and hydrophobicity considerations in bioenabling oral formulations approaches—A PEARRL review. J. Pharm. Pharmacol..

[15] Leeson P.D., Davis A.M. (2004). Time-related differences in the physical property profiles of oral drugs. J. Med. Chem..

[16] Markovic M., Ben-Shabat S., Keinan S., Aponick A., Zimmermann E.M., Dahan A. (2019). Lipidic prodrug approach for improved oral drug delivery and therapy. Med. Res. Rev..

[17] Di L., Kerns E.H. (2003). Profiling drug-like properties in discovery research. Curr. Opin. Chem. Biol..

[18] Dulsat J., López-Nieto B., Estrada-Tejedor R., Borrell J.I. (2023). Evaluation of free online ADMET tools for academic or small biotech environments. Molecules.

[19] Rutkowska E., Pająk K., Jóźwiak K. (2013). Lipophilicity—Methods of determination and its role in medicinal chemistry. Acta Pol. Pharm. Drug Res..

[20] OECD (2022). Test No. 117: Partition Coefficient (n-Octanol/Water), HPLC Method.

[21] Roman I.P., Mastromichali A., Tyrovola K., Canals A., Psillakis E. (2014). Rapid determination of octanol-water partition coefficient using vortex-assisted liquid-liquid microextraction. J. Chromatogr. A.

[22] Eadsforth C.V., Moser P. (1983). Assessment of reverse-phase chromatographic methods for determining partition coefficients. Chemosphere.

[23] Soares J.X., Santos Á., Fernandes C., Pinto M.M.M. (2022). Liquid chromatography on the different methods for the determination of lipophilicity: An essential analytical tool in medicinal chemistry. Chemosensors.

[24] Jeleń M., Pluta K., Latocha M., Morak-Młodawska B., Suwińska K., Kuśmierz D. (2019). Evaluation of angularly condensed diquinothiazines as potential anticancer agents. Bioorg. Chem..

[25] Skonieczna M., Kasprzycka A., Jeleń M., Morak-Młodawska B. (2022). Tri- and pentacyclic azaphenothiazine as pro-apoptotic agents in lung carcinoma with a protective potential to healthy cell lines. Molecules.

[26] http://swissadme.ch.

[27] https://biosig.unimelb.edu.au/pkcsm/prediction.

[28] https://www.molinspiration.com/services/logp.html.

[29] (2022). ChemDraw: ChemDraw Ultra.

[30] Bodor N., Gabanyi Z., Wong C.K. (1989). A new method for the estimation of partition coefficient. J. Am. Chem. Soc..

[31] Mannhold R., Cruciani G., Dross K., Rekker R. (1998). Multivariate analysis of experimental and computational descriptors of molecular lipophilicity. J. Comput. Mol. Des..

[32] Brooke D., Dobbs J., Williams N. (1986). Octanol:water partition coefficients (P): Measurement, estimation, and interpretation, particularly for chemicals with P > 10^5^. Ecotoxicol. Environ. Saf..

[33] Azman M., Sabri A.H., Anjani Q.K., Mustaffa M.F., Hamid K.A. (2022). Intestinal absorption study: Challenges and absorption enhancement strategies in improving oral drug delivery. Pharmaceuticals.

[34] Tsakovska I., Pajeva I., Al Sharif M., Alov P., Fioravanzo E., Kovarich S., Worth A.P., Richarz A.N., Yang C., Mostrag-Szlichtyng A. (2017). Quantitative structure-skin permeability relationships. Toxicology.

[35] Gombar V.K., Hall S.D. (2013). Quantitative structure−activity relationship models of clinical pharmacokinetics: Clearance and volume of distribution. J. Chem. Inf. Model..

[36] Poulin P., Theil F.P. (2002). Prediction of pharmacokinetics prior to in vivo studies. 1. Mechanism-based prediction of volume of distribution. J. Pharm. Sci..

[37] Mulpuru V., Mishra N. (2021). In silico prediction of fraction unbound in human plasma from chemical fingerprint using automated machine learning. ACS Omega.

[38] Sweeney M.D., Zhao Z., Montagne A., Nelson A.R., Zlokovic B.V. (2019). Blood-Brain Barrier: From physiology to disease and back. Physiol. Rev..

[39] Horde G.W., Gupta V. (2024). Drug Clearance. 20 June 2023. StatPearls [Internet].

[40] Das A., Kumar S., Persoons L., Daelemans D., Schols D., Alici H., Tahtaci H., Karki S.S. (2021). Synthesis, in silico ADME, molecular docking and in vitro cytotoxicity evaluation of stilbene linked 1,2,3-triazoles. Heliyon.

[41] Goetz G.H., Shalaeva M. (2018). Leveraging chromatography based physicochemical properties for efficient drug design. ADMET DMPK.

[42] Kaliszan R. (2007). QSRR: Quantitative Structure-(Chromatographic) Retention Relationships. Chem. Rev..

[43] Kempińska D., Chmiel T., Kot-Wasik A., Mróz A., Mazerska Z., Namie’snik J. (2019). State of the art and prospects of methods for determination of lipophilicity of chemical compounds. Trends Anal. Chem..

[44] Di L., Kerns E.H. (2008). Drug-Like Properties.

[45] Petrauskas A.A., Kolovanov E.A. (2000). ACD/Log P method description. Perspect. Drug Discov. Des..

[46] Lipinski C.A., Lombardo F., Dominy B.W., Feeney P.J. (1997). Experimental and computational approaches to estimate solubility and permeability in drug discovery and development settings. Adv. Drug Deliv. Rev..

[47] Law V., Knox C., Djoumbou Y., Jewison T., Guo A.C., Liu Y., Maciejewski A., Arndt D., Wilson M., Neveu V. (2014). DrugBank 4.0: Shedding new light on drug metabolism. Nucleic Acids Res..

[48] Ghose A.K., Viswanadhan V.N., Wendoloski J.J. (1999). A knowledge-based approach in designing combinatorial or medicinal chemistry libraries for drug discovery. A qualitative and quantitative characterization of known drug databases. J. Comb. Chem..

[49] Veber D.F., Johnson S.R., Cheng H.-Y., Smith B.R., Ward K.W., Kopple K.D. (2002). Molecular properties that influence the oral bioavailability of drug candidates. J. Med. Chem..

[50] Egan W.J., Merz K.M., Baldwin J.J. (2000). Prediction of drug absorption using multivariate statistics. J. Med. Chem..

[51] Martin Y.C. (2005). A Bioavailability Score. J. Med. Chem..

[52] Daina A., Michielin O., Zoete V. (2017). SwissADME: A free web tool to evaluate pharmacokinetics, drug-likeness and medicinal chemistry friendliness of small molecules. Sci. Rep..

[53] Lobo S. (2020). Is there enough focus on lipophilicity in drug discovery?. Expert Opin. Drug Discov..

[54] Pantaleao S.Q., Fernandes P.O., Goncalves J.E., Maltarollo V.G., Honorio K.M. (2022). Recent advances in the prediction of pharmacokinetics properties in drug design studies: A review. Chem. Med. Chem..

[55] Daina A., Zoete V. (2016). A BOILED-Egg to predict gastrointestinal absorption and brain penetration of small molecules. Chem. Med. Chem..

[56] Šegan S., Penjišević J., Šukalović V., Andrić D., Milojković-Opsenica D., Kostić-Rajačić S. (2019). Investigation of lipophilicity and pharmacokinetic properties of 2-(methoxy)phenylpiperazine dopamine D2 ligands. J. Chromatogr. B Anal. Technol. Biomed. Life Sci..

[57] Moriguchi I., Shuichi H., Liu Q., Nakagome I., Matsushita Y. (1992). Simple method of calculating octanol/water partition coefficient. Chem. Pharm. Bull..

[58] Moriguchi I., Shuichi H., Nakagome I., Hirano H. (1994). Comparison of reliability of log P values for drugs calculated by several methods. Chem. Pharm. Bull..

[59] Stanisz A. (2007). Przystępny Kurs Statystyki z Zastosowaniem STATISTICA PL na Przykładach Medycyny; Analizy Wielowymiarowe.

