# Supplementary material for: Study of the Lipophilicity and ADMET Parameters of New Anticancer Diquinothiazines with Pharmacophore Substituents
Source: Pharmaceuticals (Basel). 2024 Jun 3;17(6):725. doi: 10.3390/ph17060725 (PMC11206290; doi:10.3390/ph17060725)
Supplement: Supplementary file 1 [file pharmaceuticals-17-00725-s001.zip › pharmaceuticals-3037843-supplementary.pdf]

Study of the Lipophilicity and ADMET Parameters of New Anticancer Diquinothiazines with Pharmacophore Substituents

Daria Klimoszek [1], Małgorzata Jeleń [2,*], Małgorzata Dołowy [1] and Beata Morak-Młodawska [2]

[1] Department of Analytical Chemistry, Faculty of Pharmaceutical Sciences in Sosnowiec, Medical University of Silesia in Katowice, Jagiellońska Street 4, 41-200 Sosnowiec, Poland; d201204@365.sum.edu.pl (D.K.); mdolowy@sum.edu.pl (M.D.)

[2] Department of Organic Chemistry, Faculty of Pharmaceutical Sciences in Sosnowiec, Medical University of Silesia in Katowice, Jagiellońska Street 4, 41-200 Sosnowiec, Poland; bmlodawska@sum.edu.pl

* Correspondence: manowak@sum.edu.pl

Content:

1. Table S1. The absorption and metabolism descriptors for compounds **1 – 15**. 2
2. Table S2. The excretion and toxicity descriptors for compounds **1 – 15**. 3
3. Figure S1. The bioavailability radars for compounds **1 – 15**. 4
4. Table S3. The summary formulas, molecular weights, melting points and appearance of the tested diquinothiazines **1 – 15.** 5

Table S1. The absorption and metabolism descriptors for compounds **1 – 15**.

No. of Compound	P-glycoprotein Substrate	P-glycoprotein I Inhibitor	P-glycoprotein II Inhibitor	CYP2D6 Substrate	CYP3A4 Substrate	CYP1A2 Inhibitior	CYP2C19 Inhibitior	CYP2C9 Inhibitior	CYP2D6 Inhibitior	CYP3A4 Inhibitior
**1**	-	+	+	-	+	+	-	-	+	+
**2**	+	+	+	-	+	+	+	-	+	-
**3**	+	+	+	-	+	+	-	-	-	+
**4**	+	+	+	-	+	+	-	-	-	+
**5**	+	+	+	-	+	+	+	-	+	+
**6**	+	+	+	-	+	+	-	-	+	+
**7**	+	+	+	-	+	+	-	-	+	+
**8**	+	+	+	-	+	+	+	-	+	+
**9**	+	+	+	-	+	+	+	-	+	+
**10**	+	+	+	-	+	+	-	-	+	+
**11**	+	+	+	-	+	+	+	-	+	+
**12**	+	+	+	-	+	+	-	-	+	+
**13**	+	+	+	+	+	+	-	-	+	+
**14**	+	+	+	+	+	+	-	-	+	+
**15**	+	+	+	-	+	+	+	-	+	+

Table S2. The excretion and toxicity descriptors for compounds **1 – 15**.

No. of Compound	Renal OCT2 Substrate	AMES Toxicity	hERG I Inhibitor	hERG II Inhibitor	Hepatotoxicity	Skin Sensitisation
**1**	-	+	-	+	+	-
**2**	-	-	-	+	+	-
**3**	-	-	-	+	+	-
**4**	-	+	-	+	+	-
**5**	-	+	-	+	+	-
**6**	-	+	-	+	+	-
**7**	-	+	-	+	+	-
**8**	+	+	-	+	+	-
**9**	+	+	-	+	+	-
**10**	-	-	-	+	+	-
**11**	-	+	-	+	+	-
**12**	-	+	-	+	+	-
**13**	-	+	-	+	+	-
**14**	-	-	-	+	+	-
**15**	-	+	-	+	+	-

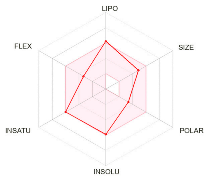

7-(2′-Diethylaminoethyl)diquino[3,2-b;3′,4′-e]thiazine
1

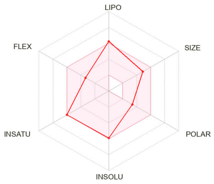

7-(2′-Diethylaminoethyl)diquino[3,2-b;6′,5′-e]thiazine
6

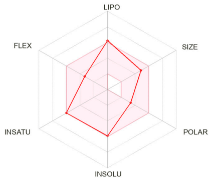

14-(2′-Diethylaminoethyl)diquino[3,2-b;8′,7′-e]thiazine
11

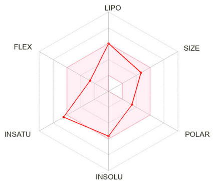

7-(3′-Dimethylaminopropyl)diquino[3,2-b;3′,4′-e]thiazine
2

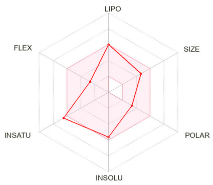

7-(3′-Dimethylaminopropyl)diquino[3,2-b;6′,5′-e]thiazine
7

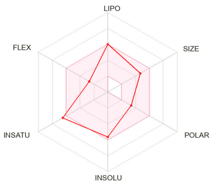

14-(3′-Dimethylaminopropyl)diquino[3,2-b;8′,7′-e]thiazine
12

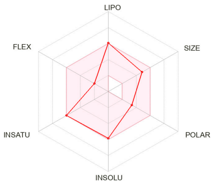

7-(1′-Pyrolidinylethyl)diquino[3,2-b;3′,4′-e]thiazine
3

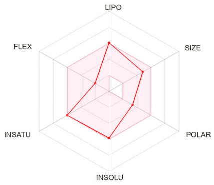

7-(1′-Pyrolidinylethyl)diquino[3,2-b;6′,5′-e]thiazine
8

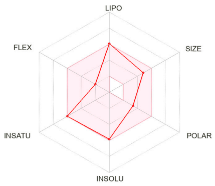

14-(1′-Pyrolidinylethyl)diquino[3,2-b;8′,7′-e]thiazine
13

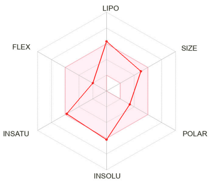

7-(1′-Piperidinylethyl)diquino[3,2-b;3′,4′-e]thiazine
4

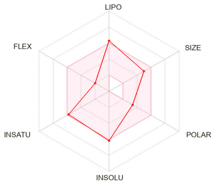

7-(1′-Piperidinylethyl)diquino[3,2-b;6′,5′-e]thiazine
9

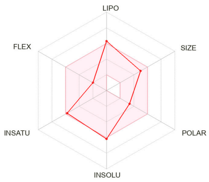

14-(1′-Piperidinylethyl)diquino[3,2-b;8′,7′-e]thiazine
14

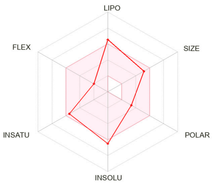

7-(1′-Methyl-2′-piperydinylethyl)diquino[3,2-b;3′,4′-e]thiazine
5

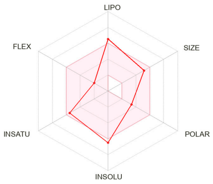

7-(1′-Methyl-2′-piperydinylethyl)diquino[3,2-b;6′,5′-e]thiazine
10

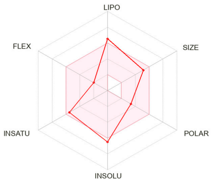

14-(1′-Methyl-2′-piperydinylethyl)diquino[3,2-b;8′,7′-e]thiazine
15

Figure S1. The bioavailability radars for compounds **1 – 15**.

Table S3. The summary formulas, molecular weights, melting points and appearance of the tested diquinothiazines **1-15**.

No. of Compound	Chem. Formula	Mol. wt.	Physical Appearance	Melting Point °C (lit. m.p. [24])
**1**	$C_{24}H_{24}N_4S$	400,50	yellow oil	-
**2**	$C_{23}H_{22}N_4S$	386,52	yellow solid	129.8 - 130.7 (130 - 131)
**3**	$C_{24}H_{22}N_4S$	398,53	yellow oil	-
**4**	$C_{25}H_{24}N_4S$	412,56	orange oil	-
**5**	$C_{26}H_{26}N_4S$	426,58	yellow solid	144.2 - 145.0 (144 - 145)
**6**	$C_{24}H_{24}N_4S$	400,50	yellow oil	-
**7**	$C_{23}H_{22}N_4S$	386,52	yellow oil	-
**8**	$C_{24}H_{22}N_4S$	398,53	yellow oil	-
**9**	$C_{25}H_{24}N_4S$	412,56	orange oil	-
**10**	$C_{26}H_{26}N_4S$	426,58	orange oil	
**11**	$C_{24}H_{24}N_4S$	400,50	yellow oil	-
**12**	$C_{23}H_{22}N_4S$	386,52	yellow oil	-
**13**	$C_{24}H_{22}N_4S$	398,53	yellow oil	-
**14**	$C_{25}H_{24}N_4S$	412,56	orange oil	-
**15**	$C_{26}H_{26}N_4S$	426,58	orange solid	143.5 – 144.8 (144 - 145)
